# Engagement and Communication Challenges for a Complex, Multi-nation Research Project: AD|ARC (Administrative Data | Agricultural Research Collection)

**DOI:** 10.23889/ijpds.v9i5.2615

**Published:** 2024-09-18

**Authors:** Freya Pryce, Sarah Lowe, Sian Morrison-Rees, Laura Madden, Paul Caskie, Racheal Loftus

**Affiliations:** 1 Welsh Government; 2 Swansea University; 3 Agri-Food and Bioscience Institute (AFBI)

The Administrative Data | Agricultural Research Collection (AD|ARC) is a complex research project involving four nations, a mixture of business-level, household-level and person-level data, and diverse partners, stakeholders and audiences. The project is creating independent but comparable Research Ready Datasets, using linked data for farm businesses, farmers and farm families in the four UK nations. The engagement and communications workstream is crucial to build trust, inform the programme of work, support and enhance scientific objectives, and deliver high-quality, relevant findings that meet stakeholder and funder expectations.

The project deployed a bespoke engagement and communications framework to meet these objectives. This presentation shares key elements of AD|ARC project's engagement and communications framework and strategy:

- Multi-region Approach: Balancing the need for a harmonised, federated resource with respecting regional differences in data, trusted research environments, and stakeholder perspectives.
- Transparency and Trust: Engaging diverse stakeholders, including farmers, researchers, and policymakers, to build trust, support and legitimacy.
- Collaborative Decision-Making: Interacting with diverse partners, (data owners, researchers, research subject representatives) to refine research questions.
- Outreach and Dissemination: A multi-method, iterative approach to raise awareness, share and promote the project.

Results are demonstrated by success in being selected as an ADR UK Flagship Dataset; integrating diverse perspectives into the creation of comparable Research Ready Datasets; consensus on a research programme; useful learning which is incorporated into strategy; and actionable evidence to inform policymaking.

The presentation will reflect on lessons learned, drawing out implications for research and public engagement that could apply to any complex research project.

